# Supplementary material for: The interactome of intact mitochondria by cross-linking mass spectrometry provides evidence for coexisting respiratory supercomplexes
Source: Mol Cell Proteomics. 2017 Dec 8;17(2):216–32. doi: 10.1074/mcp.RA117.000470 (PMC5795388; doi:10.1074/mcp.RA117.000470)
Supplement: Supplemental Data [file supp_RA117.000470_133922_0_supp_23978_fzffwf.zip › spectra_annotation/mito_DR_spectra_annotation/448-1-12-1-11-1.pdf]

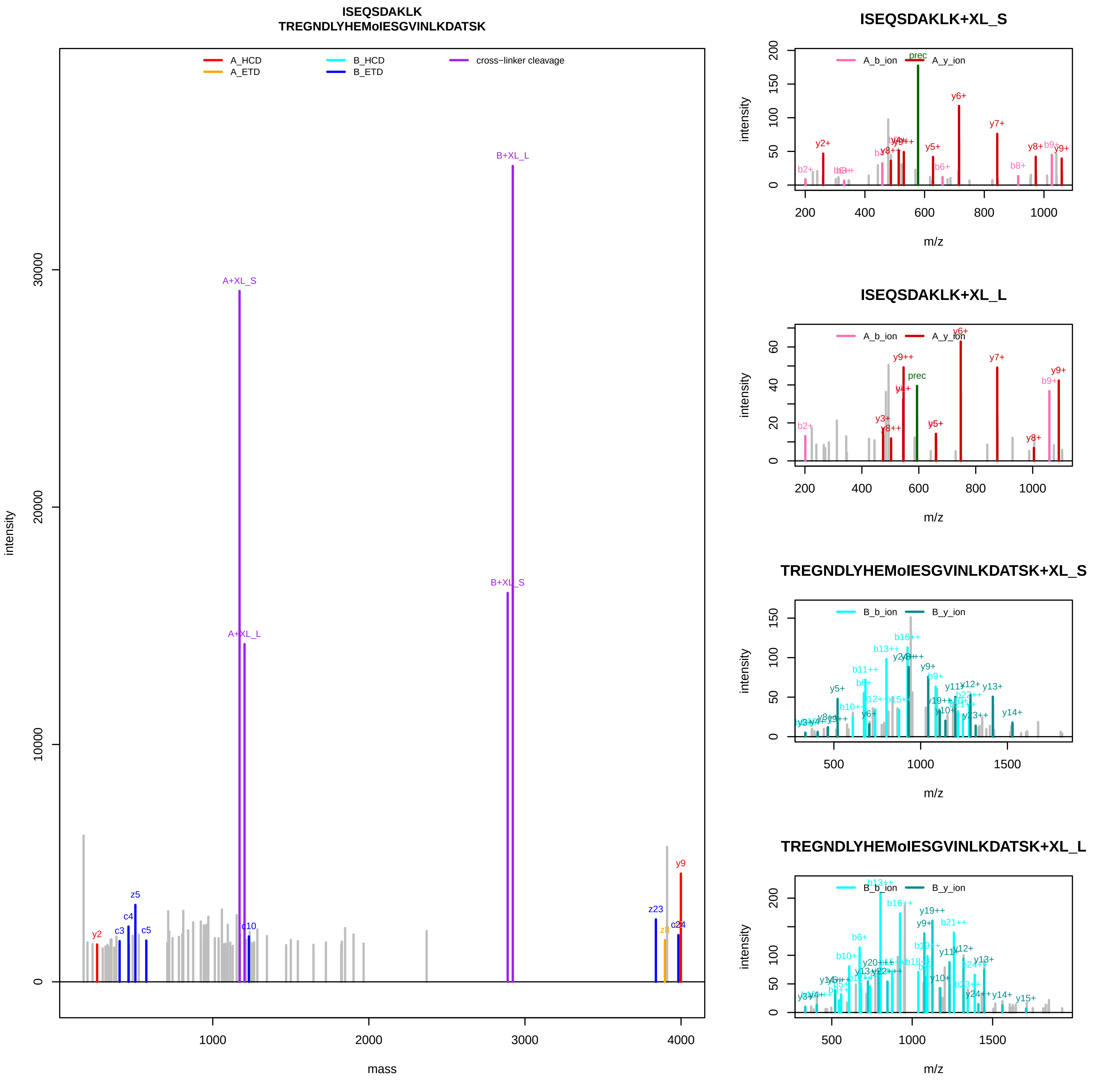
ISEQSDAKLK
TREGNDLYHEMoIESGVINLKDATSK
A_HCD
A_ETD
B_HCD
B_ETD
cross−linker cleavage
B+XL_L
A+XL_S
B+XL_S
A+XL_L
y2
c3
c4
z5
c5
c10
z23
z8
c24
y9
intensity
mass
0
10000
20000
30000
1000
2000
3000
4000
ISEQSDAKLK+XL_S
A_b_ion
A_y_ion
prec
y6+
y7+
y2+
y5+
y9+
y8+
b8+
m/z
ISEQSDAKLK+XL_L
y9++
y3+
b2+
b9+
TREGNDLYHEMoIESGVINLKDATSK+XL_S
B_b_ion
B_y_ion
b16++
b13++
b11++
y9+
b9+
y5+
y13+
y14+
TREGNDLYHEMoIESGVINLKDATSK+XL_L
b13++
b16++
y19++
b21++
b6+
b10++
y10+
y15+
